# Smartphone pregnancy apps: systematic analysis of features, scientific guidance, commercialization, and user perception

**DOI:** 10.1186/s12884-024-06959-1

**Published:** 2024-11-25

**Authors:** Michael Nissen, Shih-Yuan Huang, Katharina M. Jäger, Madeleine Flaucher, Adriana Titzmann, Hannah Bleher, Constanza A. Pontones, Hanna Huebner, Nina Danzberger, Peter A. Fasching, Bjoern M. Eskofier, Heike Leutheuser

**Affiliations:** 1 Department Artificial Intelligence in Biomedical Engineering, Machine Learning and Data Analytics Lab, Friedrich-Alexander-Universität Erlangen-Nürnberg (FAU), Erlangen, Germany; 2 Department of Gynecology and Obstetrics, Erlangen University Hospital, Friedrich-Alexander- Universität Erlangen-Nürnberg (FAU), Erlangen, Germany; 3 Department of Social Ethics, University of Bonn, Bonn, Germany

**Keywords:** Pregnancy applications, Smartphone applications, Parenting apps, Pregnant, Mobile health app, Mobile phone app, MHealth, Mobile health

## Abstract

**Background:**

Over 50% of pregnant women use pregnancy applications (apps). Some app s lack credibility, information accuracy, and evidence-based clinical advice, containing potentially harmful functionality. Previous studies have only conducted a limited analysis of pregnancy app functionalities, expert involvement/evidence-based content, used commercialization techniques, and user perception.

**Methods:**

We used the keyword “pregnancy” to scrape (automatically extract) apps and app information from Apple App Store and Google Play. Unique functionalities were derived from app descriptions and user reviews. App descriptions were screened for evidence-based content and expert involvement, and apps were subsequently analyzed in detail. Apps were opened and searched for used commercialization techniques, such as advertisements or affiliate marketing. Automated text analysis (natural language processing) was used on app reviews to assess users’ perception of evidence-based content/expert involvement and commercialization techniques.

**Results:**

In total, 495 apps were scraped. 226 remained after applying exclusion criteria. Out of these, 36 represented 97%/88% of the total market share (Apple App Store/Google Play), and were thus considered for review. Overall, 49 distinct functionalities were identified, out of which 6 were previously unreported. Functionalities for fetal kick movement counting were found. All apps are commercial. Only 15 apps mention the involvement of medical experts. 10.3% of two-stars user reviews include commercial topics, and 0.6% of one-/two-/three-/five stars user reviews include references to scientific content accuracy.

**Conclusion:**

Problematic features and inadequate advice continue to be present in pregnancy apps. App developers should adopt an evidence-based development approach and avoid implementing as many features as possible, potentially at the expense of their quality or over-complication (“feature creep”). Financial incentives, such as grant programs, could support adequate content quality. Caregivers play a key role in pregnant individuals’ decision-making, should be aware of potential dangers, and could guide them to trustworthy apps.

## Background

The use of pregnancy applications (apps) is widespread among pregnant women. Literature reports that over 50% of all pregnant women use or have used at least one smartphone app for pregnancy in the past, based on studies in Australia, China, and Ireland [1–3]. This demand is matched by severe supply: The number of pregnancy apps surpasses the market for all other medical topics [4], with the most popular apps featuring over 10 million installations and 1.5 million user reviews. Pregnancy apps offer a wide range of features and functionalities. Some focus on individual pregnancy aspects (such as the search for a baby name), while others offer encompassing bundles, aiming to cover all potential pregnancy facets [5].

Previous studies have investigated numerous aspects and relationships of pregnancy apps, such as their use by expectant mothers, overall perception [6–8], as well as the impact on medical caregivers and in general [9, 10]. Pregnancy apps are mainly used for information seeking, particularly for health and fetal development information, and are perceived as useful [2]. Healthcare providers and family/friends are the most frequently used source for pregnancy information, followed by the internet and mobile phone apps [11].

Earlier pregnancy app reviews revealed that only a few apps offer an overall high quality based on an assessment using the Mobile Application Rating Scale (MARS) tool, particularly concerning pregnancy-focused nutrition information [12, 13]. They furthermore showed that most apps lack desired features [14, 15] and that literature or science boards were seldomly cited/stated [14, 16]. The quality and correctness of information vastly differ between apps. An earlier review identified a lack of evidence-based clinical advice to guide women experiencing decreased fetal movements [17]. References to scientific sources or evidence-based medicine are often missing, particularly for controversial topics such as exercise or alcohol intake [18, 19]. Some apps deliver mostly medically accurate information [20]. Pregnancy app users state lack of credibility as a major weakness of pregnancy apps, and a structured analysis found information credibility to be overall low [8]. App users state that gold standard and evidence-based information was preferred to “just doctors’ opinions” [2].

The regular use of antenatal care apps, particularly disease-screening functions, is associated with a higher rate of antenatal depression [21]. Incorrect information can also lead to raised anxiety [2], which is particularly relevant in the presence of “Cyberchondria”, an escalation of self-enforcing online searches associated with health anxiety [22–24]. The use of lifestyle apps is associated with smartphone addiction, particularly in female users [25].

Consequently, pregnancy apps have an impact on pregnant women, their pregnancy journey, and involved healthcare professionals. Apps offer the potential to increase patient awareness, participation in their health journey, and to empower patients’ autonomy. At the same time, among other factors, they pose new challenges for caregivers advising pregnant women, particularly with respect to false information, functionality lacking clinical evidence, and Cyberchondria [9, 10, 26].

Prior pregnancy app reviews are subject to several limitations: They have only reviewed less than 10 apps [15, 19, 20], solely focused on overall quality using app assessment scores such as MARS or APPLICATIONS [12–15], focused on behavior-change techniques [12, 15, 27], or provided only broad information on features and functionalities [8, 14, 19]. Analysis of expert involvement has been limited so far [18, 19]. User perception was analyzed through user surveys, focus groups, and interviews in the past [2, 8, 28]. To the best of our knowledge, app reviews have not yet been analyzed for this purpose, despite the large amount of available user reviews. Previous work has, furthermore, called for studies examining a larger number of apps [19].

This work contributes to the existing body of knowledge by (i) being the first to cover and review the majority of pregnancy apps in practical use based on objective criteria, (ii) providing a comprehensive list of pregnancy app features for future medical analysis and discussion, (iii) analyzing scientific rationales and employed commercialization and monetization techniques, and (iv) being the first to investigate pregnancy app user perception based on app reviews using natural language processing (NLP). NLP is a collection of computational techniques to process and analyze text and human language in an automated way [29].

These aspects are important to gain a better understanding of the overall pregnancy app ecosystem as well as previously outlined problems, to deliver better care for pregnant women.

**Fig. 1:**
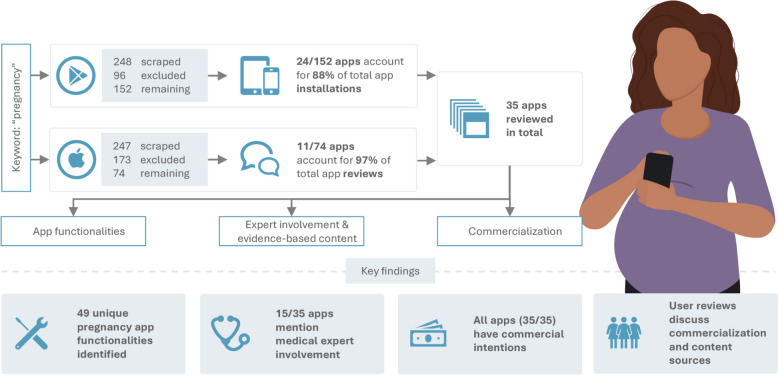
Graphical abstract of this work, which aims to identify key app functionalities, the use of evidence-based content and commercialization techniques. The focus of this abstract is on the methods and key findings of this work. Own illustration

A graphical abstract of this paper is shown in Fig. 1.

## Methods

We first present details on the employed web scraping (information extraction) as well as review methodology in this chapter. Following that, the chosen method regarding functionality, expert involvement, commercialization, and user perception is described.

### Information extraction

Web scraping is a method to automatically extract information from websites using software. We used Node.js and the Node.js-packages “google-play-scraper” [30] and “app-store-scraper” [31] to retrieve a list of pregnancy apps from the Apple App Store and Google Play store. Both libraries are conceptually similar. For both stores, the keyword “pregnancy” was used together with the “fullDetail” option, with the language set to English (“en”) and the country default of United States (“us”). We used a broad keyword (“pregnancy”) with a subsequent manual exclusion step (see next Subsection) to avoid missing relevant applications based on inadequate keyword selection. As the Apple App Store library only returns app IDs, the “AppStoreScraper” Python package [32] was used to retrieve additional details.

Both stores limit the number of scrapable apps. At the time of this study’s conduction, this limit was approximately 200 apps for the Apple App Store and 250 apps for Google Play. It was not possible to retrieve offsets, e.g. to retrieve apps 250 to 500. Consequently, we were forced to limit each query to 200/250 apps. The search results returned by Google Play and Apple App Store are not deterministic. The results change between searches and may be influenced by several factors, including location, (personal account) history, general trends, location, operating system, and time. To minimize the impact of this feature, we used no accounts (logged out) as well as different accounts, with and without the browser private mode, used searches at different time of days and accessed the services from two IP addresses (personal and via our university’s virtual private network), and two different operating systems (Microsoft Windows and Apple macOS). We considered an app as eligible if it appeared in any of the queries.

### App selection, eligibility and exclusion criteria

We only included apps that were primarily focused on pregnancy companioning and encompassed more than one key functionality, as they are the target of this review. We thus excluded apps that focused solely on baby photo editing, fertility/period advice/tracking, baby name search, weight and diet control/information, baby monitoring, exercise for pregnancy, music for pregnancy, due date or contraction trackers, postpartum information, drugs and symptoms information, pregnancy tests, entertainments/music as well as virtual pregnancy games or baby face generators and ultrasound guides/assistance apps for caregivers. We included both free and paid apps in our analysis.

We applied a Pareto-principle [33] oriented approach to identify a minority of apps that account for the largest number of installations. The Pareto principle states that roughly 80% of the effects result from 20% of the causes. In this sense, a small number of pregnancy apps account for the largest user base. Overall, detailed usage data is unavailable from both app stores. Google Play only publishes installation numbers in broad steps (e.g. 10,000,000, 5,000,000, 1,000,000, 500,000 installations), while the Apple App Store does not provide any installation numbers, only user review counts. We thus used these metrics (installation numbers for Google Play, review counts for Apple App Store) to identify the apps with the highest impact on the respective app ecosystems.

We did not remove duplicate apps, as apps from the same manufacturer/with the same name may have different functionalities for different operating systems. App features and content behind paywalls (in-app purchases) were not analyzed in our review.

### Functionality assessment

Two reviewers independently assessed the functionalities of eligible apps: A master’s student in artificial intelligence as well as a doctoral candidate in computer science and digital health with a background in mobile health app development. Reading through the app descriptions, each reviewer identified and clustered similar features. Results were documented in tabular form, using app names and features to create an app-feature grid. After creating the initial lists, both reviewers discussed the identified features, adding, renaming, separating, or combining features, ultimately establishing a final list of app features. This was necessary as features are often named differently, fluent in nature (not exactly distinguishable) or interwoven with other functionalities. Based on the final feature list, one of the two reviewers re-reviewed all app descriptions to ensure consistency.

### Expert involvement and evidence-based content

We screened the descriptions of all previously selected apps. If the descriptions included terms such as *articles*,* tips*,* support from experts*, and *information reviewed by doctors* or *cooperated with organizations*, these apps were included for a second analysis step. The respective expert claim in the app description (e.g. “[App name] gives you trusted information backed by experts”) was noted. In the second analysis step, both the website of the individual apps as well as the app itself were opened and screened for detailed information about the previously stated expert inclusion, i.e. the name of involved physicians or organizations. We categorized apps into the categories “own team”, “organizations”, “physicians/midwife/nurse by name”, “organization” and “no information”. We furthermore reviewed included information articles and recorded whether the authors of individual articles were listed.

### Commercialisation

Different monetization options exist for app manufacturers. These include, but are not limited to user payments, advertisements, affiliate marketing, sales channels, or user data selling [34, 35]. We screened the app descriptions for references of subscription-based or one-time payment plans and related possibilities. Afterwards, all apps were opened and manually analyzed. We noted the service provided in exchange for payment (only ads removed, additional content). Furthermore, the apps were screened for monetization channels, grouped in in-app stores for physical goods (e.g. purchase of pregnancy equipment), presence of affiliate marketing links (e.g. to Amazon), payment for medical services (e.g. chat with doctors or answering of medical questions) and the presence and type of advertisements (Google AdSense, other external services, own app shop).

### Gamification

We noted whether the apps used gamification elements (e.g. to earn points or coins by interacting with the apps and how these coins can be used).

### User perception

We used all available app reviews of the identified apps to assess user perception of scientific ground and commercialisation. To gather the apps, Python was used for web scraping. For Apple App Store apps, we used the app_store_scraper [32] package to gain user review data. For Google Play, respectively, the google_play_scraper [36] package was utilized. We used only US-reviews (country=’us’ setting) and did not include a data limit or a maximum number of reviews.

We separately assessed scientific ground and commercialisation related reviews. For both cases, a random sample of 10,000 reviews was drawn for both Google Play and Apple App Store. These 20,000 reviews were then manually screened for references towards scientific ground or commercialisation, and the respective keywords were extracted. We identified several terms and statements. Commercial analysis terms, for example, included terms such as “*upgrad*[ing]” the App, “*pay*[ing] for” it, “*cost*”, “$”, “sell[ing]” things, “*premium* version” or “*full* version”, ”lots of ads”, and ”*commercial* [videos]”. For the scientific analysis, exemplary terms are “asking questions and getting answers from *doctors*”, “I wish it was kept closer to *scientific evidence* based info”, “I should say, *expert* are too good”, “checked by a *medical expert*”, “I did not appreciate the recent *pseudoscientific* articles being peddled via this app”. Afterwards, each identified term was enriched with related synonyms using dictionary.com [37] to broaden the search scope.

Separately for commercialization and scientific terms, we used the matcher() function of spaCy Python’s package [38] to extract all reviews including words on either of the word lists.

We relied on (user review) sentiment analysis to assess the perception of users towards scientific ground and commercialization. This is a generalizable and intuitive approach towards user perception, particularly concerning a large number of reviews.

To determine sentiment, we relied on the *Valence Aware Dictionary and sEntiment Reasoner* (VADER) [39], provided by the NLTK Python package [40]. VADER is a rule-based approach to sentiment analysis, developed for social media text, which we deemed was most applicable in the context of user reviews. NLTK’s VADER implementation returns a *compound* score, which represents the sentiment of the underlying text, ranging from − 1 (very negative) to + 1 (very positive).

## Results

### Included apps

Differences between the searched results across all web scraping requests were subtle. Out of 248 initially identified apps on Google Play, 96 were excluded based on our exclusion criteria. Out of the remaining 152 apps, we selected those with at least 1 million installations, as they represent 88% of all installations, resulting in 24 apps. On the Apple App Store, 74 out of 247 apps remained after applying exclusion criteria, and the top 11 most reviewed app account for 97% of all reviews. We thus analyzed 35 identified apps (24 from Google Play and 11 from Apple App Store) in the following steps. 8 out of 35 apps were included in both app stores. None of the apps required a payment for initial download.

### Functionality

In the next step, the functionalities of each included app were assessed. We overall identified 49 distinct features, which are listed in Table 1. Features are not exclusive, and some are similar in nature.

**Table 1:** List of identified functionalities with number of occurrences, in descending order of occurrence count

Functionality	Count	Description
Pregnancy Progress Information (“Week by week”)	28	Regular reminders, notifications and information about current pregnancy progress, e.g. information about current physiological changes in the respective pregnancy week.
General Medical Information	24	General medical pregnancy information.
Physiological Data Tracker	24	Track physiological parameters such as weight, diet, activities, skin, mood, sleep, blood pressure and nutrition.
Due Date Calculator/Countdown	20	Countdown to due date.
Information Section	18	General information articles, tips, news and videos.
Contraction Timer	17	Allows measurement of contractions throughout labor.
Calender	15	Any arbitrary calendar function. This may include an appointment organizer, important dates during pregnancy or development steps.
Nutrition Advice	15	Nutrition tips and recipe information.
Baby Kick Counter	14	Counter to track fetal kicks.
Belly Photos	14	Functionality specifically targeted at “belly photos”.
Name Finder	14	Search for baby names, often including information on meaning, origin, and theme.
Shopping List	13	Product suggestions, recommendations and reviews.
Discussion Forums	11	Integrated discussion forums.
Symptom Tracker	11	Recording and tracking of symptoms throughout the pregnancy.
Breastfeeding Advice	10	Breastfeeding advice and information.
Timeline/Milestone	10	Baby development tracking, often including milestones, e.g. by charts or timelines.
3D Fetal Growth Model	9	Interactive 3D models showing baby development.
Activity Advice	9	Health and pregnancy exercise information, such as yoga or mother-baby activities.
Appointment Organizer	9	Keeps track of appointments with physicians/midwifes. May also integrate with Android/iOS calendars.
Baby’s Growth Tracker	9	Enter measurement data to track growth, e.g. for comparison against World Health Organization averages.
Hospital Bag (Checklist)	8	Advice for packing a hospital bag, often in checklist form.
Photo/Video Storage/Editing	8	May include diary, album as well as editing functions (stickers, frames, backgrounds).
Social Sharing	8	Ability to share photos, e.g. in social media.
Birth Plan Assistant	7	Advice on and/or creation of a birth plan, i.e. outline preferences during labor/delivery.
Ovulation Tracker	7	Functionality to assist in fertility planning.
Lifestyle (Relax/Sleep) Advice	6	Information and suggestions on lifestyle related matters (e.g. tips for relexation/sleeping).
Medication Advice	6	Information on medication and medication safety during pregnancy or breastfeeding.
Compare Fetus’s Size	5	Vivid comparison of current fetus size, e.g. to fruits or animals, according to current pregnancy week.
Examination Information	5	Information about typical pregnancy examinations.
External Data Backup/Sharing	5	Export and/or synchronization functions, e.g. as booklet, PDF, Excel-Files, E-Mail, Airdrop, Social Media Sharing, Google Drive iCloud, Dropbox.
Wearable Integration	5	E.g. Apple Health Kit integration
Baby Movement Tracker	4	Record when movements of the baby occurred.
Ultrasound Album	4	Functionality specifically targeted at ultrasound images.
Baby Feeding/Diaper/Sleep Tracker	3	Tracks sleep, feeding and diaper changes of the baby.
Expert Assistance	3	Access (e.g. via chat or email) to experts to answer pregnancy and parenting related questions.
Multilinguality	3	Support for multiple languages.
Partner/Family Account	3	Integration of partner/family into app functions (e.g. allow partner to see pregnancy progress).
Vaccination Tracker	3	Track vaccinations of mother and/or child.
Artificial Intelligence Technology	2	Any mention of “AI technology” (e.g. smart contraction tracker, pregnancy prediction based on menstrual history)
Birth Classes	2	Health coaching, personalized content about your benefits, and health programs
Community Games	2	Community games, some including the ability to win prize money.
Customization/Theming	2	Customizable app theme/color scheme.
Fashion Advice	2	Information on maternal and baby clothing.
Frequently Asked Questions	2	List of frequently asked questions, may include “intelligent” search tools.
Health Report	2	Regular (e.g.) monthly health report about pregnancy progress, personal situation and events.
Medical History Log and Tracker	2	Medication history and medication tracker.
Health Alert	1	Data feedback/real-time health alerts based on tracked symptoms.
Knowledge Tests	1	Quizzes about pregnancy and childbirth.
Offline Access	1	App usable without internet access.

### Expert involvement and evidence-based content

Another aim of this work was the analysis of expert involvement and evidence-based content. 15 out of 35 apps mentioned for example *articles*,* tips*,* supports from experts*, *information reviewed by doctors*, or *cooperated with organizations* in their descriptions. They were thus selected for in-depth review in the next step. Out of these 15 apps, 11 mention physicians, nurses or midwives by name, 4 mention organizations (such as the “United Nations Population Fund in the area of reproductive health”), 2 mention an “own team”, and for 1 app no details could be found, despite such a statement in the app description. 3 apps were assigned to two categories, as they for example stated both an organization and a caregiver by name.

The results of the perception analysis for scientific keywords are outlined in Table 2. The percentage of reviews containing scientific keywords is similar across all star ratings. Apple App Store reviews discuss scientific aspects more frequently. The compound of ratings increases from 3 star to 4 star ratings, while 1, 2 and 3 star ratings as well as 4 and 5 star ratings are similarly ranged.

**Table 2:** Occurrence of keywords and average compound of app reviews related to evidence-based content and scientific keywords

Rating	Apple app store: reviews containing scientific keywords	Google play: reviews containing scientific keywords	Apple app store: Average compound of reviews containing scientific keywords	Google play: average compound of reviews containing scientific keywords
1 star	0.6%	0.2%	0.144	-0.114
2 stars	0.6%	0.3%	0.303	0.074
3 stars	0.6%	0.1%	0.249	-0.033
4 stars	0.5%	0.1%	0.436	0.285
5 stars	0.6%	0.2%	0.411	0.341

### Commercialisation

Similar to the analysis of expert involvement and evidence-based content, we aimed to investigate the employed commercialization and monetization techniques of pregnancy apps. Pregnancy apps use various monetarization approaches. With respect to purchases, app upgrades or additional offerings, 18 out of 35 apps offer in-app purchases, ranging from 0.99$ up to 100.00$ per item. 10 apps provide additional features in exchange for payment. Most (22/35) apps show advertisements. In some apps, these can be removed for payment (9/35).

Affiliate marketing (e.g. to Amazon products) was identified in 15 out of 35 apps. 2 apps offer own online shops. In one of these two cases, a referral program was present, where app users could earn a commission of 1%, e.g. by sharing products via social media or instant messaging. 2 out of 35 apps offered “points” or “coins” for replying on articles, regular usage or friend referrals/invites. These points were offered to be exchanged for unspecified “rewards” or “presents”. Notably, 3 out of 35 apps offer additional medical consultancy services for payment, e.g. an online chat with caregivers. All apps used at least one of the aforementioned commercialization techniques.

The results of the perception analysis for commercial keywords are outlined in Table 3. Commercial keywords are much more frequent in reviews with lower star ratings (10% and 5.8%) compared to higher star ratings (0.6% and 0.4%). Apple App Store reviews overall contain commercial keywords more frequently. For reviews with commercial keywords, the average rating compound is lowest for 1 star reviews and highest for 5 star reviews.

**Table 3:** Occurrence of keywords and average compound of app reviews related to commercialization

Rating	Apple app store: reviews containing commercial keywords	Google play: reviews containing commercial keywords	Apple app store: average compound of reviews containing commercial keywords	Google play: average compound of reviews containing commercial keywords
1 star	10%	5.8%	0.073	-0.024
2 stars	10.3%	6.5%	0.168	0.052
3 stars	7.9%	3.4%	0.198	0.127
4 stars	2.5%	0.8%	0.320	0.256
5 stars	0.6%	0.4%	0.487	0.431

## Discussion

This work aimed to analyze pregnancy app features, expert guidance and content trustworthiness, commercialization, and their user perception. For this purpose, we reviewed app descriptions, performed in-depth searches, and used NLP on user reviews. This section will discuss key findings in the respective areas, derive overall implications, provide insights for clinical practice, limitations of this work, and lastly provide links for future research.

### Principal results

#### Similar core features across reviewed apps

Our results demonstrate that pregnancy apps offer a wide range of different functionalities. Functionality to track pregnancy progress, medical pregnancy information, and trackers for various physiological data are most common. In related work, Scott et al. [19] aggregated ten key features from ten analyzed apps. Two of these (healthy lifestyle development and adoption during and after pregnancy, information on skin health) were not explicitly identified in our review. Womack et al. [18] identified nine key functionalities, with “week by week development” (45/48 apps in [18]), “pictures of baby development” (38/48 apps in [18]), and “weight tracker” (32/48 apps in [18]) as most commonly found. “Postpartum depression screen” was not explicitly identified as a unique feature in our review. O’Donnell et al. [20] analyzed and coded content delivered by two apps throughout usage. They identified fetal development, prenatal care, pregnancy symptoms, and maternal well-being as key topics. A study issuing a questionnaire to a Chinese audience previously found that monitoring fetal development was by far (436/535 participants) the leading reason for pregnancy app usage [2]. Most apps identified by us covered this key use case.

#### New features are being introduced to pregnancy apps

We used a fine-grained approach to functionality identification. This allowed us to identify several functionalities that were not named in literature yet, particularly “Integration of wearables and fitness trackers” (5/35), “AI technology” (2/35), “smart health alerts” (1/35), “health reports” (2/35), “birth classes” (2/35), and “expert assistance” (3/35). These indicate that ubiquitous health, machine learning, and telemedicine are being adapted and brought to consumers.

#### Inaccurate information and features without scientific ground continue to be present in pregnancy apps

Functionalities for kick (14/35) and movement counting (4/35) continue to be present in apps. Previous work found that one-third of apps offer kick counters [17]. There is no clear evidence for performing such counts [41]. Apps moreover were previously found to present information for fetal movement stimulation that is inadequate or discouraged in clinical practice guidelines [17].

Incorrect, inaccurate, and conflicting information is a problem in pregnancy apps. Information about nutrition, exercise, hair dye, immunization, maternal fetal movement, weight tracking, and breastfeeding was particularly affected [13, 15, 18]. Studies, trials and related evidence is often not or insufficiently cited [14, 15, 18, 19].

#### Minority of apps are developed in cooperation with caregivers

Only 15 out of 35 apps in our review stated cooperations with caregivers or such organizations. Content articles only featured the name of caregivers in two cases. This is similar to prior research, where only four out of 10 reviewed apps involved health professionals in development and evaluation [19], and only three out of 51 apps noted affiliations and funding sources [13]. A longitudinal study found that although functionality and usability of apps increased within a two year period, content credibility did not [15]. Our findings are in line with the present body of knowledge.

Large apps typically employ some kind of board of experts. We could, however, not find information how this board of experts is involved. In addition to the integration of experts, previous research, studies and projects can provide valuable assistance to developers. For example, they can offer insights into user requirements, best practices and lessons learned [42–45].

#### Some users are aware of inaccurate information

We analyzed the perception of “scientific” app aspects through app reviews. Apple App Store reviews (0.5–0.6%, see Table 2) more frequently include “scientific” keywords compared to Google Play reviews (0.1–0.3%). No clear trend is visible between individual star ratings. Some users thus seem to be aware of potential quality and content issues. When reviewing individual statements, users frequently mention the “reliability” of an app. A minority of reviews directly mention evidence-based content, for example “Deleted this app after I received a recommendation to inject myself and my unborn child with an experimental biologic product, directly followed by a recommendation to not eat ice cream (…)” or “I am so frustrated with the ludicrous medical claims this app makes.”. Previous work demonstrate as well that pregnant women are overall worried about lack of scientifically validated content in pregnancy apps, some specifically asking for a gold standard or evidence-based guide [2].

#### Commercialization is widespread

All reviewed apps in our study are of commercial nature. Advertisements are the leading revenue channel (22/35), followed by in-app purchases (18/35), affiliate links (15/35), online consultation services (3/35), and own online shops (2/35). This confirms previous results of a similar study, where all apps were commercially developed, and 4/10 apps offered the purchase of app upgrades or linked to online shops for baby-related products [14]. The study moreover found that some apps were directly linked or at least affiliated with shops or manufacturers for nipple creams, bras and breastmilk substitutes; all apps lacked funding details transparency.

#### Commercialization is discussed in user reviews

The percentage of reviews containing commercial keywords is relatively high (0.4-10.3%, see Table 3), particularly compared to the previously discussed scientific keywords. Notably, commercial keywords are much more present in unfavorable reviews (Apple App Store: 10% vs. 0.6%, Play Store: 5.8% vs. 0.4%). As negative reviews are usually phrased in more negative voice, it is unsurprising that the compound is lowest in one-star reviews, and highest in five-star reviews. To name some aspects, individual reviews criticize excessive paywalls/pricing, the selling of personal information, as well as advertising placements/mechanisms such as pop-ups. Some apps are stated to be unusable without payment.

We initially found the percentage of reviews containing commercial keywords surprising, whereas apps are usually linked to commercial goals. It seems that a larger number of users are confronted with the commercial nature of pregnancy apps. In some instances, they are even criticized. On the other hand, commercialization is a central and important aspect for application developers and providers, who usually pursue commercial goals.

#### Implications of the nontrivial ecosystem of pregnancy apps

The relationship between app functionalities, information accuracy, scientific guidance, commercialization, and users is nontrivial. As outlined before, as of now, some apps contain potentially dangerous patterns for becoming mothers. This may be due to incorrect information or features and users’ adherence to it [13, 15, 17, 18, 41], but can also secondarily lead to increased, unjustifiable anxiety [46]. Implementing as many features and content as possible (“feature creep”) can thus not be the answer to the development of pregnancy apps. At the same time, our analyzed reviews indicate that some users demand specific features, including questionable ones such as kick counters, in their (negative) reviews. These reviews are detrimental for app developers, as they influence both users in their download decisions as well as app store search algorithms. From this point of view, there is an incentive for app developers to adapt inadequate functionalities.

While the prevalence of incorrect information has been shown in previous works, it is unclear how this incorrect information is being generated. Possible reasons include unfamiliarity with the domain and literature, lack of expert guidance, selection of wrong experts, incorrect expert information, controversial guidelines and gold standards, and finally, inadequate funding. Not all users are willed to pay for apps [2], which can be confirmed by the large number of negative reviews addressing pay-for content and paywalls.

It must be acknowledged that, while apps try to assist the users in solving their problems (in this case providing pregnancy information and assisting throughout their health journey), app developers pursue commercial goals to keep supporting their product. The pregnancy app market is larger than that for any other medical topic [4], and attracts respective (fierce) competition.

Related works state regulation by an authoritative agency to combat some of the above problems [5, 47]. This could be a government agency, or the app store operator. In the first case, a quick implementation is unlikely, as apps are present in a large number of countries, consequently including dozens of respective agencies. On the other hand, such a regulation or oversight is particularly relevant in countries where no structured prenatal care otherwise exists, and users potentially use pregnancy apps as the only or key source of information – one of the key advantages of mobile health. At the same time, small companies and developers could be disadvantaged by regulation, as it could imply a large overhead. In either case, a direct fix of the existing situation is unlikely due to the political processes required to regulate it at different levels.

Implementing additional revenue sources that reward trustworthy content and expert collaboration can be another way to increase (medical) app quality. Government agencies or health insurances could offer monetary incentives for app developers if certain quality guidelines are met. Such quality guidelines could include certain functionalities, but also content requirements (e.g. peer-reviewed information). The disadvantage of such an approach is the effort for such collaboration, likely involving significant bureaucracy. In contrast, advertisements or affiliate programs are much faster to integrate into apps and provide nearly instant monetary results.

#### Implications for clinical practice

Not only pregnant women, but also care providers have to be aware of the omnipresence of pregnancy apps [1–3]. This also includes the awareness of incorrect content. The use of digital helpers is unlikely to disappear. Therefore, users should be informed about inadequate features, such as kick counters, as well as potential hazards in information articles. A likely more practical alternative is the recommendation of specific apps after prior screening by the individual medical professional. This is associated with a one-time effort. Professional associations, but also hospitals, could support individual caregivers by developing recommendations. As outlined before, cooperation with specific app developers, or the development of an own app could serve as an alternative and offer potential for both sides. In either case, caregivers should not only review apps for content accuracy but can also see whether specific functions (e.g. journaling) can be integrated into their medical care.

#### Limitations

Several limitations apply to our work. We extracted information from the app stores using web scraping libraries. For this purpose, we set the language parameter to English and location parameter to United States. Although we used different IP addresses to mitigate the effect of geographic location on search results, our research team was located in Germany. This location can have an impact on the web scraping results and thus the list of apps assessed in this work. Outside the findings reported in this work, we examined the effect of the location (country) in an exploratory and unstructured manner. We found the overall changes in results to be negligible, and therefore have not included this approach here. In contrast to prior work, we focused on an approach using app descriptions to analyze app functionality. Functionalities differ between apps, and separations between functionalities are not precisely possible in all cases but instead are a result of discussion between the reviewers. Therefore, the results are subject to a certain degree of uncertainty. For example, the “baby kick counter” and “baby movement tracker” features are similar. Nevertheless, some apps explicitly state they record “kicks” (kick counter), while others promote the tracking of “kicks, jabs, pokes, rolls, or swishes”. We have therefore distinguished between these features. We only assessed app descriptions but did not perform an in-depth analysis by downloading and opening apps. Apps were not excluded if they were present in both Apple App Store and Google Play, as apps can offer different functionalities for each platform, and we were unable to ensure that the apps were the same in terms of features. This approach is also chosen in related works.

We did not use the Mobile Application Rating Scale (MARS) [48], as we were specifically interested in pregnancy app functionalities. Although the scale covers a *functionality* subsection, a specific analysis of individual functionalities is not provided. Several previous publications already assessed pregnancy apps using this scale [12, 13, 15, 27], and we thus chose a different focus for this work. We only assessed the presence of references to scientific sources or experts but did not check the accuracy or validity of the provided/referenced content.

The use of app reviews for our means is subject to several limitations. Reviews do not necessarily reflect a product’s true quality [49], and can be faked, e.g. to artificially boost an app’s ranking [50]. Our NLP approach is basic, as it is mostly based on keyword extraction, and offers room for future work.

#### Contribution

This work contributes to the existing body of knowledge in several ways. Overall, it is the first work to systematically assess and cover most of the English-language pregnancy market based on app downloads, which makes it the most comprehensive review to date. A detailed analysis of individual app functionalities has previously not been conducted, and this work provides a detailed list of respective functionalities. It confirms previous literature results on scientific guidance and commercialization, showing that these results apply to the overall pregnancy app market. Lastly, we are the first to provide results from a NLP-based analysis of app store reviews with respect to both commercial and expert guidance aspects.

#### Future work

Five key areas particularly profit from future work. First, the provided list of functionalities forms the basis for an in-depth analysis of the medical rationale and potential implication on women’s health for each individual function. Newly emerging functionalities such as the integration of machine learning algorithms as well as wearable devices are of particular interest. This can include an analysis of the use of personal data such as user profiles. Second, the use of advanced NLP techniques can provide new insights on user perception in all areas covered by this review (functionalities, commercialization, expert inclusion/evidence-based content). Third, the development of a list of recommended apps for the use for clinical practitioners or the development of a pregnancy app that uses an “evidence-first” approach. Fourth, guidelines for app developers on the reporting of collaborations, evidence-based work, and information sources should be developed. Lastly, the development of a regulatory framework for fostering app development with evidence-based content, taking into account the commercial realities of the app market.

## Conclusions

This work aimed to analyze pregnancy app features, expert guidance and content trustworthiness, commercialization, and their user perception, with an emphasis on an extensive list of pregnancy app functionalities. We identified six features and offerings that were previously not reported in literature. All apps reviewed in this work appear to follow commercial interests. A minority involves experts or includes information on scientific validity. Problematic features and inadequate advice continue to be present in apps.

App development should follow a two-track user need and evidence-based development approach: User-centered design ensures that key user needs are addressed while basing this on scientific evidence, reconsidering every functionality, while refraining from feature creep. Users can include pregnant individuals, but also their partners, family, and friends. Promoting an evidence-based approach (opposed to maximum features) can offer a competitive advantage over peer pregnancy apps. Financial incentives, such as grant programs, supporting content quality and expert collaboration may be able to alleviate these issues in the long term and could serve as an alternative to regulation.

Caregivers play a key role in the selection of pregnancy apps. Caregivers should be aware of potential dangers from the use of these apps. Selecting a suitable app as personal recommendation may be helpful for patient guidance. The development of an “official” app focusing on limited core functionalities, developed in close cooperation with official institutions, may be able to overcome these pitfalls in the future.

## Data Availability

Individual participants’ data that underlie the results reported in this article and a data dictionary defining each field in the set are available to investigators whose proposed use of the data has been approved by an independent review committee for work. Proposals should be directed to weima@sdu.edu.cn to gain access, data requestors will need to sign a data access agreement. Such requests are decided on a case by case basis.

## Abbreviations

App: Application
MARS: Mobile application rating scale
NLP: Natural language processing
VADER: Valence aware dictionary and sEntiment reasoner
